# Highly Stable and Non‐Volatile Optoelectronic Synapses for Sensing, Memory, and Computing Enabled by a Deep‐Potential Floating Gate

**DOI:** 10.1002/advs.78038

**Published:** 2026-09-27

**Authors:** Shuo Lei, Shichen Zhang, Zhipeng Zheng, Miao Cai, Shun Tang, Xuguang Guo, Yiming Zhu

**Affiliations:** ^1^ National Key Laboratory of Terahertz Perception and Communication (TPCL) University of Shanghai for Science and Technology Shanghai China; ^2^ Shanghai Key Lab of Modern Optical Systems Terahertz Technology Innovation Research Institute and Engineering Research Center of Optical Instrument and System Ministry of Education University of Shanghai for Science and Technology Shanghai China; ^3^ Shanghai Institute of Intelligent Science and Technology Tongji University Shanghai China

**Keywords:** metal floating gate, neural network, non‐volatile memory, optoelectronic synaptic transistor

## Abstract

Optoelectronic synapses integrating optical sensing and memory functions are promising building blocks for neuromorphic visual systems. Here, an optoelectronic synaptic transistor based on a MoS_2_/h‐BN/Au heterostructure with Au as the floating gate is demonstrated. Benefiting from the high work function of the metal floating gate (MFG), the device exhibits efficient non‐volatile charge storage with a large memory window of 97.6 V and a stored charge density of 7.0 × 10^12^ cm^−2^. Under optical stimulation, the device shows pronounced wavelength‐dependent synaptic plasticity. The device exhibited data retention exceeding 3000 s and cycling endurance over 1600 programming/erasing cycles. Separately, reproducible programming/erasing operation was observed after 200 days of storage under ambient conditions. In particular, 450 nm illumination generates high‐energy photocarriers in the MoS_2_ channel, which more effectively modulate the charge state of the MFG through carrier transfer across the h‐BN barrier, leading to stable long‐term potentiation behavior. More importantly, grounding the MFG suppresses the non‐volatile optical synaptic response, highlighting the dominant contribution of floating‐gate charge storage over interfacial trapping effects. Using experimentally measured weight‐update characteristics, a hardware‐aware neural‐network simulation achieves 98.4% test accuracy on MNIST handwritten‐digit classification. This work highlights the potential of the MFG architecture for integrated optoelectronic memory and neuromorphic vision applications.

## Graphical abstract

A deep‐potential metal floating gate integrates electrical programming, optical partial erasure, and synaptic weight modulation in a MoS_2_/h‐BN/Au transistor. The device provides a 97.6 V memory window, retention beyond 3000 s, endurance over 1600 cycles, and reproducible operation after 200 days of ambient storage. Experimentally derived conductance updates enable 98.4% MNIST recognition, highlighting multimodal sensing‐memory‐computing integration.

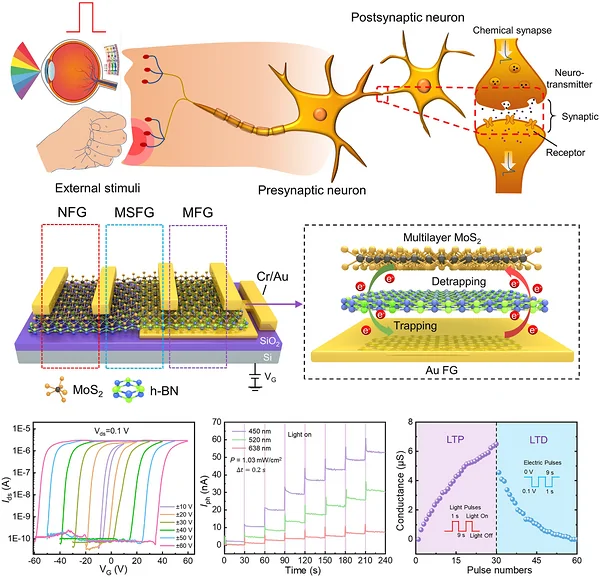

## 1 Introduction

Neuromorphic computing systems inspired by the human brain have emerged as promising candidates for overcoming the energy consumption and data‐transfer bottlenecks of conventional von Neumann architectures [1, 2, 3]. Among various neuromorphic hardware platforms, optoelectronic synaptic devices capable of integrating sensing, memory, and signal processing functionalities have attracted significant attention for artificial visual systems and in‐sensor computing [4, 5]. However, the rapidly increasing demand for visual data processing still poses significant challenges for conventional computing architectures in terms of memory efficiency and power consumption [6, 7]. Therefore, developing optoelectronic synaptic devices with integrated sensing‐memory‐computing capabilities has become an important research direction for next‐generation neuromorphic visual systems [8].

In particular, two‐dimensional (2D) transition metal dichalcogenides (TMDCs) exhibit great potential for optoelectronic synapses owing to their atomic‐scale thickness, excellent electrostatic tunability, and strong light‐matter interactions [9, 10, 11]. Moreover, van der Waals (vdW) heterostructures constructed by stacking different 2D materials can effectively avoid lattice‐matching constraints while enhancing carrier transport and interfacial coupling properties [12, 13]. Benefiting from these unique advantages, various 2D‐material‐ based photodetectors, memories, and field‐effect transistors have been widely developed for multifunctional integrated electronics [14, 15]. Numerous efforts have utilized TMDCs and their vdW heterostructures to enhance memory performance, such as InSe/h‐BN/graphene (Gr) [16], ReS_2_/h‐BN/Gr [17, 18, 19], BP/h‐BN/Gr [20], SnS_2_/h‐BN/Gr [21], PtS_2_/h‐BN/Gr [22], MoTe_2_/h‐BN/Gr [23] and MoS_2_/h‐BN/Gr [24, 25, 26], demonstrating robust non‐volatile functionality. Nevertheless, the relatively low work function of Gr (∼4.6 eV) and its sensitivity to environmental molecules often lead to Fermi level shifts induced by charge‐trapping states or chemical adsorption. This instability results in fluctuations of the potential well depth and significant charge leakage, resulting in negative influence on data retention [27]. While alternative floating gate materials, including organic films [28, 29, 30], TaN [31], Pt [32] and metal nanoparticles [33], have been explored, achieving stable, long‐term charge storage in vdW architectures remains challenging.

To address these limitations, using a metal floating gate (MFG) layer with a higher work function, such as Au (∼5.1 eV), offers a promising solution to reduce the leakage of storage carriers [34]. This approach constructs a more stable potential well between the tunneling layer (h‐BN) and the blocking layer (SiO_2_), thereby enhancing retention, increasing the current ON/OFF ratio between programmed and erased states, and mitigating charge leakage [35]. Such devices are highly applicable as electronic memory elements within neuromorphic systems. However, current MFG devices remain predominantly limited to electrical modulation. Synaptic devices that emulate biological visual systems with pronounced optoelectronic synergistic responses remain an important gap to be bridged.

In this work, we demonstrate a highly stable and non‐volatile optoelectronic synaptic field effect transistor (FET) enabled by a deep‐potential MFG architecture. By integrating a high work‐function Au nanofilm with a MoS_2_/h‐BN heterostructure, our design creates a deep potential well intended to mitigate volatile charge leakage, a significant challenge frequently encountered in conventional 2D synaptic devices. The thermally evaporated Au nanofilm serves as a robust charge‐storage layer, while the high‐quality h‐BN layer acts as a tunneling barrier. Due to the deep potential well and high density of states of the MFG architecture, the device delivers an exceptionally large memory window of 97.6 V. Moreover, the device exhibits data retention exceeding 3000 s and cycling endurance over 1600 programming/erasing cycles. Reproducible programming/erasing operation is also observed in a separate measurement performed after 200 days of ambient storage. Through synergistic electrical and optical modulation, we achieve multilevel storage states with high distinguishability and temporal stability, significantly broadening the dynamic range for synaptic weight tuning. Integrating these experimental weight‐update characteristics into a simulated artificial neural network (ANN) yields a peak training accuracy of 99.9% and a test recognition rate of 98.4% for the Modified National Institute of Standards and Technology (MNIST) handwritten digit classification task. These results not only validate the superiority of the MoS_2_/h‐BN/Au architecture for sensing‐memory integration but also provide an important way for developing reliable, high‐precision neuromorphic vision processing platforms.

## 2 Results and Discussion

**FIGURE 1:**
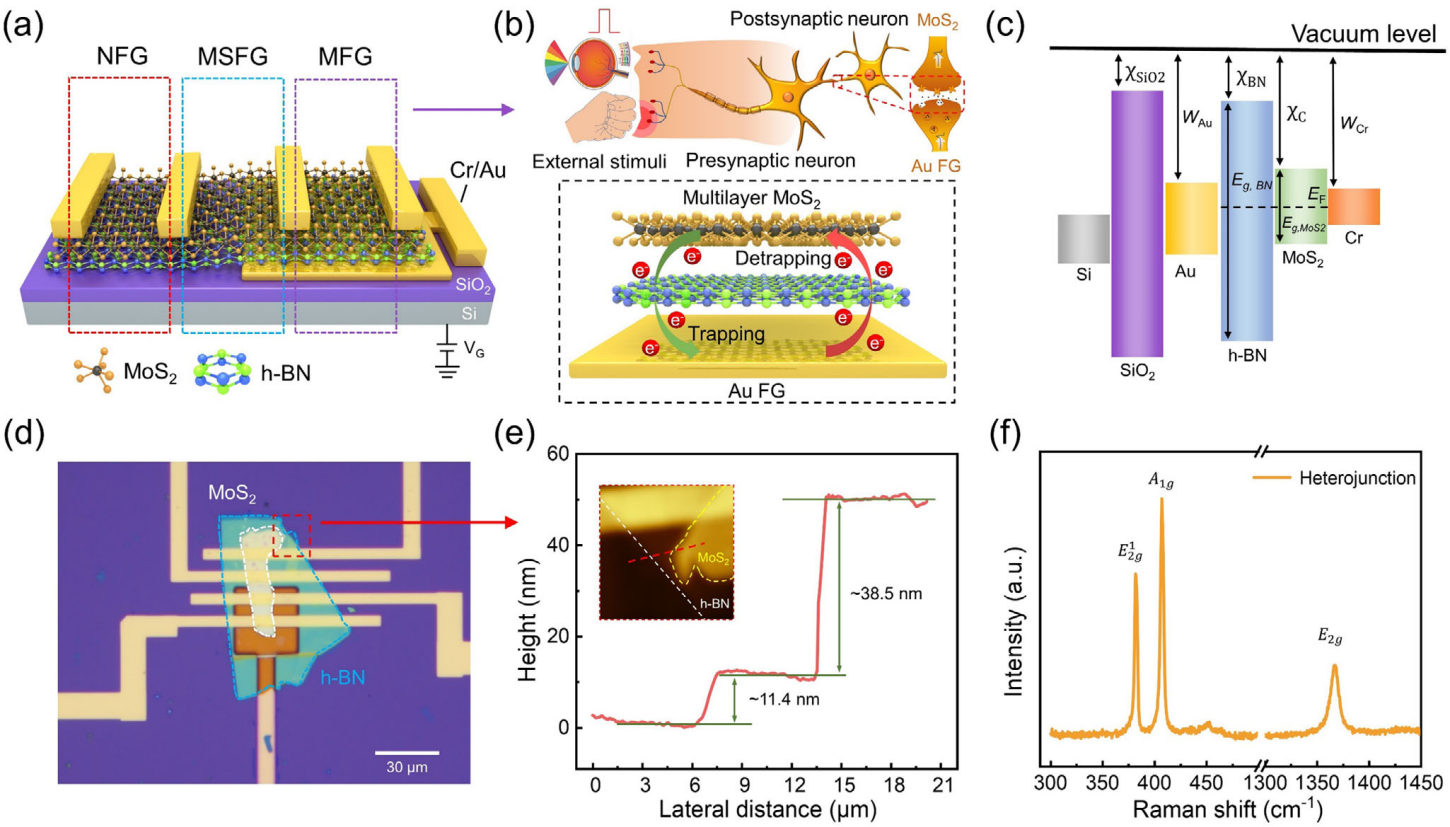
The MoS_2_/h‐BN/Au optoelectronic synaptic FET. (a) Three‐dimensional schematic of the MFG‐FET. (b) Schematic illustration of the biological synapse and the mechanism of synaptic behavior in the MFG‐FET. (c) Energy band alignments of the MoS_2_/h‐BN/Au heterostructure. (d) Optical microscopy image of the fabricated MFG‐FET. (e) AFM image and thickness characterization of MoS_2_/h‐BN. (f) Raman spectrum of the MoS_2_/h‐BN/Au heterostructure.

Figure 1a schematically illustrates the three‐dimensional architecture of the MoS_2_/h‐BN/Au optoelectronic synaptic FET. Detailed fabrication protocols are provided in the Experimental Section and Figure S1. The device comprises a multilayer MoS_2_ channel, an underlying h‐BN tunneling dielectric layer, and a thermally evaporated MFG layer on a SiO_2_/p+Si substrate. By defining specific electrode configurations, the device is divided into three functional regions: the MFG, metal semi‐floating gate (MSFG), and no‐floating gate (NFG) configurations. As depicted in Figure 1b, the device is engineered to emulate the synaptic plasticity of biological neural networks. External stimuli, applied as either electrical or optical pulses, serve as presynaptic signals, while the corresponding conductive states within the MoS_2_ channel represent the postsynaptic response. The underlying physical mechanism is strictly governed by the MFG‐related charge‐trapping dynamics. Upon electrical or optical excitation, carriers tunnel across the h‐BN potential barrier, undergoing trapping and de‐trapping processes between the multilayer MoS_2_ and the MFG. This well‐controlled charge transfer process endows the device with robust non‐volatile memory behaviors, directly facilitating the emulation of bidirectional synaptic weight updates, namely long‐term potentiation (LTP) and long‐term depression (LTD).

The energy band alignments of Cr, MoS_2_, h‐BN, and Au are illustrated in Figure 1c. Here, *W*_Cr_ = 4.5–4.6 eV [36] and *W*_Au_ = 5.1 eV [37] represent the work functions of Cr and Au, respectively. Additionally, $\chi_{\mathrm{Mo}S_{2}}$= 3.5–3.8 eV [38] and χ_h‐BN_ = 2–2.3 eV [39] denote the electron affinities of MoS_2_ and h‐BN, respectively. The corresponding bandgaps are *E*_g, MoS2_ = 1.2 eV and *E*_g, h‐BN_ = 5.9 eV [40]. Based on the vacuum level‐alignment model, the nominal electron‐confinement barrier at the Au/h‐BN interface is estimated to be *W*_Au_ −  χ_h − BN_ =  2.8 − 3.1 eV. Because the barrier (4.2 eV) on the SiO_2_ side is higher, the effective depth of the Au floating‐gate potential well is approximately 2.8–3.1 eV, without considering interfacial effects. The large bandgap of h‐BN establishes a robust physical barrier that impedes spontaneous charge leakage in the absence of an external driving force, thereby guaranteeing the non‐volatile stability of the memory states. Concurrently, the Schottky barrier height formed at the MoS_2_/Cr interface strictly determines the carrier injection efficiency. Other high‐work‐function metals may provide similar electron confinement. Au is selected for its high work function, high density of states, chemical stability, and fabrication compatibility.

The optical micrograph (Figure 1d) explicitly presents the spatial stacking of the constituent layers. The dashed lines are the boundaries of the mechanically exfoliated h‐BN layer (blue region) and the overlying MoS_2_ channel (white dashed region). To precisely obtain the material thicknesses, atomic force microscopy (AFM) was employed. As shown in the height profile (Figure 1e), the multilayer thicknesses of the h‐BN and MoS_2_ flakes are approximately 11.4 and 38.5 nm, respectively. The inset AFM morphological image corroborates a highly planar heterostructure surface with good interfacial contact. Such a high crystal quality is paramount for minimizing interfacial scattering and enhancing carrier mobility within the channel.

Figure 1f presents the Raman spectrum acquired in the heterostructure region. Two phonon peaks of the multilayer MoS_2_ are distinctly resolved: the in‐plane vibrational $E_{2g}^{1}$ mode positioned at 382 cm^−1^, and the out‐of‐plane vibrational *A*_1*g*_ mode located at 407 cm^−1^. Furthermore, the characteristic *E*_2*g*_ phonon mode of h‐BN, corresponding to its in‐plane atomic displacement, is unambiguously identified at 1368 cm^−1^. These sharp and well‐defined Raman signatures further corroborate the perfect crystalline quality of the constituent two‐dimensional materials within the fabricated heterojunction.

**FIGURE 2:**
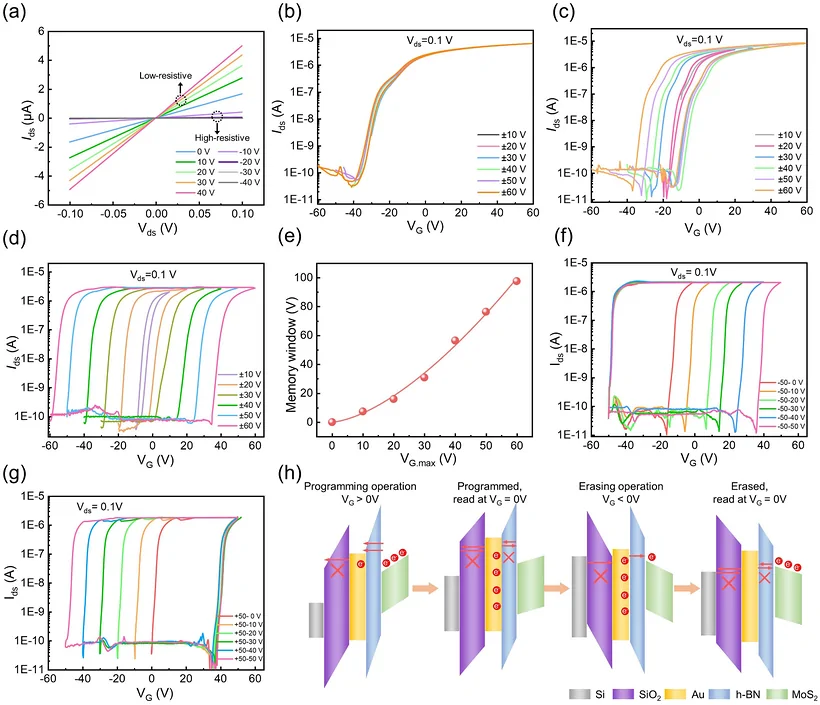
Electrical characterization of non‐volatile memory based on the MoS_2_/h‐BN/Au MFG‐ FET. (a) Output characteristics of the MFG‐FET. (b–d) Transfer characteristics under bidirectional *V*_G_ sweeps for the NFG, MSFG, and MFG devices, respectively. A distinct memory hysteresis is clearly observed for the MFG device. (e) The memory window width as a function of gate voltage sweep range. The *I*_ds_‐*V*_G_ transfer curves by bidirectionally sweeping the gate voltage (f) from the fixed −50 V to different positive values and (g) from different negative voltages to the fixed +50 V. (h) Schematic energy band diagrams elucidating the charge trapping and tunneling processes corresponding to the programming, read in programmed state, erase, and read in erased state of the MFG‐FET.

As shown in Figure 2, in order to elucidate the non‐volatile charge storage characteristics of the MFG, the electrical and memory properties of the device are systematically evaluated with different gate configurations. The output characteristics (*I*_ds_‐*V*_ds_) of the MFG device under different back gate voltages are depicted in Figure 2a. The linear *I*–*V* curves at different back gate voltages indicate the formation of near‐ideal ohmic contacts between the source/drain electrodes (Cr/Au) and the multilayer MoS_2_ channel. Additionally, as *V*_G_ is swept from −40 to +40 V, the device exhibits a pronounced field‐effect modulation of the channel conductance from high‐resistance states to low‐resistance states. The output characteristics of the NFG and MSFG devices at different back gate voltages show similar behavior (Figure S2), which shows that the ohmic contacts are not influenced by the MFG and the back‐gate condition.

To explicitly identify the physical origin of charge trapping, the transfer characteristics of the NFG, MSFG, and MFG devices were systematically measured under bidirectional *V*_G_ sweeps (Figure 2b–d). For the NFG device (Figure 2b), only a negligible hysteresis exhibits even under a large gate sweep of ±60 V, indicating an exceptionally low density of intrinsic defects within the MoS_2_ channel and at the MoS_2_‐h‐BN interface. The negative threshold gate voltage of about −40 V indicates that the MoS_2_ channel is n‐type doping. In the MSFG configuration (Figure 2c), a moderate memory window emerges as the sweep range increases, which is primarily attributed to the fact that the localized charge trapping only modulates a limited portion of the entire channel. In a clear contrast, the MFG device (Figure 2d) demonstrates a massive and highly tunable memory window (Δ*V*). The results reveal that the MFG layer dominates the carrier trapping and de‐trapping processes, and the MFG device exhibits the most pronounced memory performance, characterized by the tunable and wide hysteresis window and the large on‐off ratio of the source‐drain current. Based on the above quantitative comparison, the MFG configuration was selected as the optimal platform for all the subsequent investigations, including synaptic plasticity simulation and integrated sensing‐memory‐computing demonstrations.

Under a ±60 V bidirectional gate voltage sweep, the deep potential well of the floating gate layer due to the high work function of MFG enable the efficient injection and robust confinement of a high density of carriers. To investigate the effect of h‐BN dielectric layer thickness on the memory characteristics, several contrast devices with varying thicknesses (∼19 nm and ∼40 nm) were fabricated, and as illustrated in Figure S3, the memory behaviors were evaluated. The bidirectional‐sweep transfer characteristics reveal that while all devices display hysteresis, but the memory window widths shrink significantly with increasing the thickness of h‐BN. This reduction is primarily attributed to the increased tunneling barrier width imposed by the thicker dielectric layer, which substantially increases the threshold voltage of Fowler‐Nordheim (FN) tunneling of carriers.

The electrical parameters of the MoS_2_/h‐BN/Au MFG FET were quantitatively evaluated using the standard capacitance model. The gate capacitance per unit area *C_g_* ​ is primarily determined by the SiO_2_ dielectric layer and can be approximated (without considering the fringe electric field) as [34],

(1) $$C_{g}=\frac{\varepsilon_{0}\varepsilon_{s_{i}O_{2}}}{d_{S_{i}O_{2}}}$$

where ε_0_ is the vacuum permittivity (8.85 × 10^−14^ F/cm), $\varepsilon_{\mathrm{Si}O_{2}}$= 3.9, and $d_{\mathrm{Si}O_{2}}$= 300 nm [41]. This yields a capacitance value of *C*_g_ ≈ 1.15 × 10^−8^ F/cm^2^. Owing to the much larger thickness of the SiO_2_ layer compared to that of the h‐BN dielectric, the contribution of h‐BN to the total capacitance is negligible.

The charge density stored in the MFG was estimated using the memory window Δ*V*, according to,

(2) $$D=\frac{\Delta V\cdot C_{g}}{e}$$

where *e* is the elementary charge (1.602 × 10^−19^ C). For a memory window of Δ*V*  =  97.6 V, the stored charge density *D* ≈ 7.006 × 10^12^ cm^−2^. This indicates efficient charge trapping capability of the MFG layer.

The field‐effect mobility μ of the device was extracted from the linear regime of the transfer characteristics using [42],

(3) $$\mu =\frac{L}{W}\;\cdot \frac{1}{C_{g}V_{\mathrm{ds}}}\cdot \frac{dI_{\mathrm{ds}}}{dV_{G}}$$

where *L* is the channel length, *W* is the channel width, *V*_ds_​ is the drain‐source voltage, and $\frac{dI_{\mathrm{ds}}}{dV_{G}}$ represents the slope of the linear part of transfer characteristics of the device. The mobility of the device derived using Equation (3) and the transfer curve is about 135.3 cm^2^V^−1^s^−1^.

Furthermore, the carrier concentration in the MoS_2_ channel was evaluated using,

(4) $$n_{e}=\frac{C_{g}\left(V_{G}-V_{\mathrm{th}}\right)}{e}$$

where *V*_G_ is the applied gate voltage and *V*_th_ is the threshold voltage. Using *V*_G_ = +60 V and the experimentally extracted threshold voltage (*V*_th_ = −54.01 V), the effective gate overdrive voltage is 114.01 V, leading to a carrier concentration of *n_e_* ≈ 8.2 × 10^12^ cm^−2^. This high carrier density is consistent with the strong n‐type conduction behavior of MoS_2_ and the effective electrostatic modulation induced by the MFG. Notably, the comparable magnitude between the stored charge density and the channel carrier concentration further confirms the dominant role of the MFG in modulating the device conductance.

As shown in Figure 2e, the memory window increases almost monotonically with the maximum value of the applied back gate voltage (*V*_G, max_). This indicates the excellent tuning capability of the gate voltage on charge trapping in the MFG layer. Figure 2f,g further identify the continuous tunability of the value of *V*_th_. As the positive gate sweep amplitude of *V*_G_ increases, the value of *V*_th_ undergoes a progressive positive shift (Figure 2f). Conversely, increasing the negative sweep amplitude induces a corresponding negative shift of *V*_th_ (Figure 2g). This phenomenon elucidates the dynamic, electric‐field‐driven charge transfer processes between the MoS_2_ channel and the MFG. These experimental results clearly demonstrate that the charge trapping and de‐trapping, and then the memory window can be precisely controlled by the back gate voltage.

Figure 2h illustrates the underlying mechanisms of charge trapping and de‐trapping in the MFG‐FET. Upon the application of a positive gate pulse (*V*_G_ > 0 V), a sufficiently strong electric field is established across the tunneling layer, enabling the FN tunneling of electrons from the n‐type MoS_2_ channel to the MFG layer across h‐BN layer [43]. The electrons captured by the MFG deplete the majority carriers in the MoS_2_ channel and result in a low‐conductance programmed state. Due to the deep potential well established by the high work function of Au, these trapped electrons are robustly confined even after the back‐gate voltage pulse is removed. Conversely, a negative gate pulse (*V*_G_< 0 V) triggers the erasure process, where the reversed electric field drives the FN back‐tunneling of electrons from the MFG into the MoS_2_ channel. This re‐injection process restores the carrier density in the channel, returning the device to a high‐conductance state, as verified at a reading bias of *V*_G_ = 0 V.

In typical non‐volatile memory operations, the channel is in the high (low) conductance state after an erasing (programming) pulse, which is defined as state ‘1’ (‘0’). To determine the threshold programming/erasing voltages required for stable switching, we characterized the dynamic current response under varying back‐gate pulse amplitudes (Figure S4). Transient measurements were conducted at *V*_ds_ = 0.1 V (fixed in the following measurements). Back gate voltage pulses were applied sequentially, with the readout current *I*_ds_ monitored during the pulse intervals (*V*_G_ = 0 V). As illustrated in Figure S4a,b, the device exhibits distinct memory windows with a channel current ON/OFF ratio of approximately 10^5^ under the back gate voltage pulse amplitudes of 50 and 20 V, respectively. This performance indicates that a back gate voltage of 20 V provides a sufficient electric field to efficiently drive charge tunneling and trapping within the MFG layer. Conversely, reducing the pulse amplitude to 10 V (Figure S4c) results in a degradation of the ON/OFF ratio to the 10^4^ order. This attenuation suggests that the electric field strength at *V*_G_ = 10 V fails to effectively open the FN tunneling channel, leading to suboptimal charge‐injection efficiency and a reduced logic state distinction. Based on the voltage‐dependent ON/OFF ratio, the back gate voltage of 20 V was identified as the optimal operating bias to balance switching contrast and power consumption. Figure S4d demonstrates three consecutive memory cycles at this gate voltage: an erase pulse of ‐20 V (1 s) resets the device to an *I*_ds_ of 10^−6^ A, while a subsequent programming pulse of +20 V (1 s) switches the channel to a 10^−10^ A state. The sharp, reproducible transitions and the absence of significant state drift confirm the robust switching determinism and operational reliability of the device under low‐voltage conditions.

**FIGURE 3:**
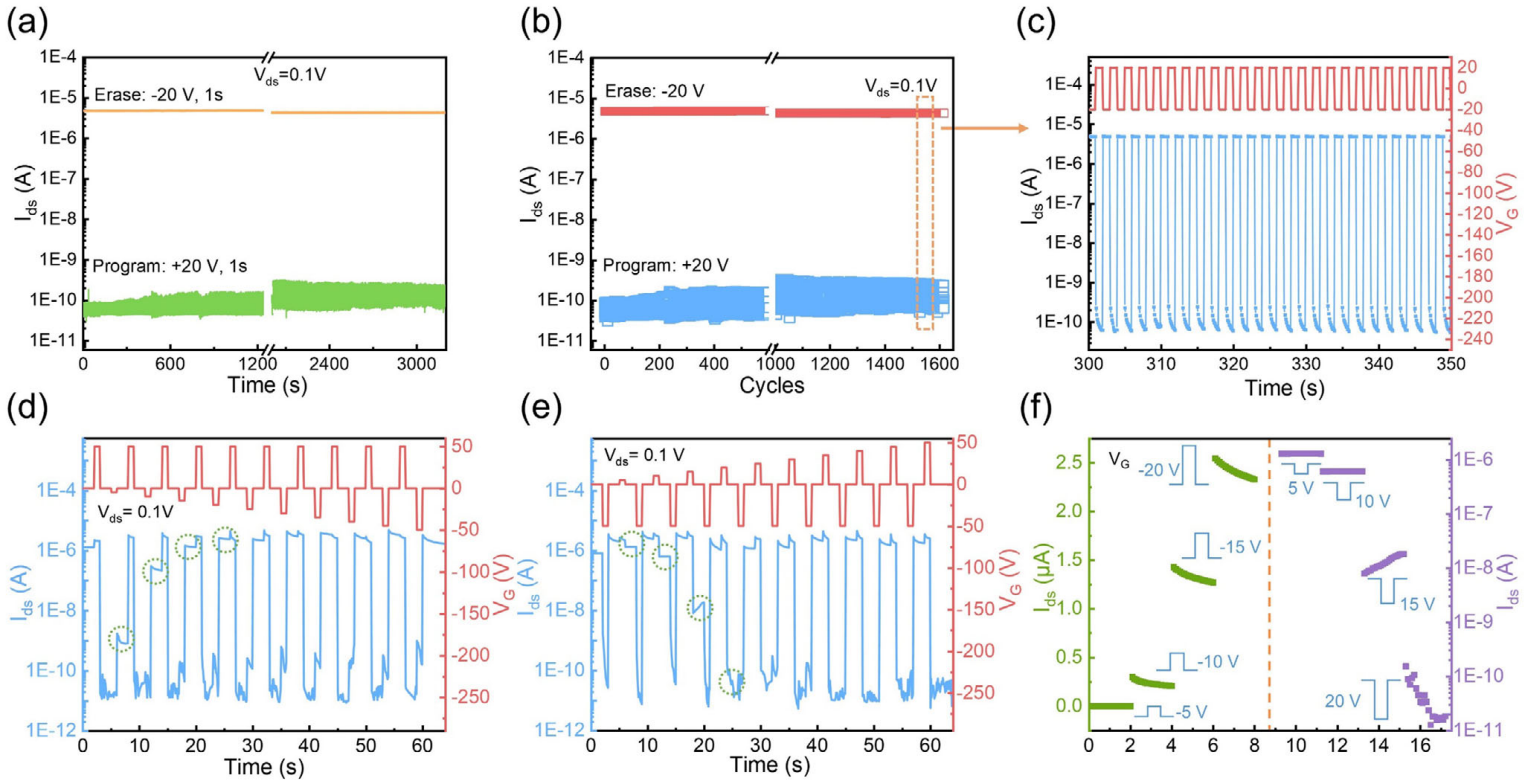
Characterizations of non‐volatile memory and emulated neuromorphic synaptic plasticity in the MFG‐FET. (a) Data‐retention characteristics of the programmed and erased states measured for more than 3000 s after single +20 V programming and −20 V erasing waveforms with a nominal duration of 1 s. The states were read at *V*_ds_ = 0.1 V. (b) Cycling endurance of the programmed and erased states over more than 1600 consecutive programming/erasing cycles. (c) Enlarged temporal trace corresponding to the selected cycling region in (b), showing repeated switching between the two current states. Pulse‐amplitude‐dependent erasing (d) and programming (e) transients recorded under varying negative and positive gate voltage amplitudes, respectively. (f) Multilevel storage capability. Under gate pulse modulation ranging from −20 V to +20 V, the channel exhibits discrete and highly distinguishable conductance states.

To systematically evaluate the electrical reliability of the MFG‐FET, data retention, cycling endurance, and ambient‐storage stability were examined as three distinct reliability metrics. As shown in Figure 3a, after programming and erasing with +20 and ‐20 V gate pulses, respectively, with a duration of 1 s, the two current states remained clearly distinguishable for more than 3000 s at *V*_ds_ = 0.1 V. The endurance performance is demonstrated through repeated programming and erasing of the fully floating gate memory cell (Figure 3b), indicating that the ON/OFF ratio remains at a high value of approximately 10^5^ even after more than 1600 cycles, with no significant degradation. The enlarged real‐time memory state (Figure 3c) exhibits a stable transition between the high‐ and low‐conductance states.

Ambient‐storage stability of the device was evaluated. After 200 days of storage under laboratory ambient conditions, the device retains reproducible programming/erasing functionality by applying 0.02 s gate‐voltage pulses, producing an ON/OFF ratio of approximately 10^5^ (Figure S5). This post‐storage measurement evaluates the device aging and is not a 200‐day data‐retention test.

To further examine the field‐assisted charge‐transfer mechanism, the bidirectional transfer characteristic was analyzed using a history‐dependent electrostatic model in which the charge transfer between the floating gate and the channel is determined by the capacitive coupling and the feedback FN tunneling. The numerically calculated bidirectional transfer loop reproduces the principal features of the experimental hysteresis (Figure S6). Moreover, the vertical *I‐V* data between the floating gate and the channel exhibit an approximately linear relationship between ln(*I/V^2^*) and 1*/V* in the range of 6.52‐10.28 V (*R* ^2^ = 0.9807; Note S1 and Figure S7), consistent with the FN tunneling.

With the aid of the exceptional electrical reliability of this MFG‐FET, we further explored the key synaptic plasticity behavior observed in biological neural networks. Figure 3d shows the results of continuous pulse measurements under different erase pulse peak voltages. It can be observed that as the absolute value of the erase pulse peak voltage increases, the current for state ‘1’ increases significantly. When the erase pulse peak exceeds 20 V, the increase in the current for state ‘1’ tends to saturate. Similarly, Figure 3e shows the results of continuous pulse measurements under different programming pulse peak voltages. When the programming pulse amplitude exceeds 20 V, the decrease in the current for state ‘0’ also tends to saturate. As shown in Figure 3f, by precisely controlling the back gate amplitude (from 5 to 20 V), the number of carriers entering the MFG layer is precisely determined, thereby achieving step‐like, multi‐steady‐state conductance levels in the MoS_2_ channel. For a detailed analysis, see Figure S8.

**FIGURE 4:**
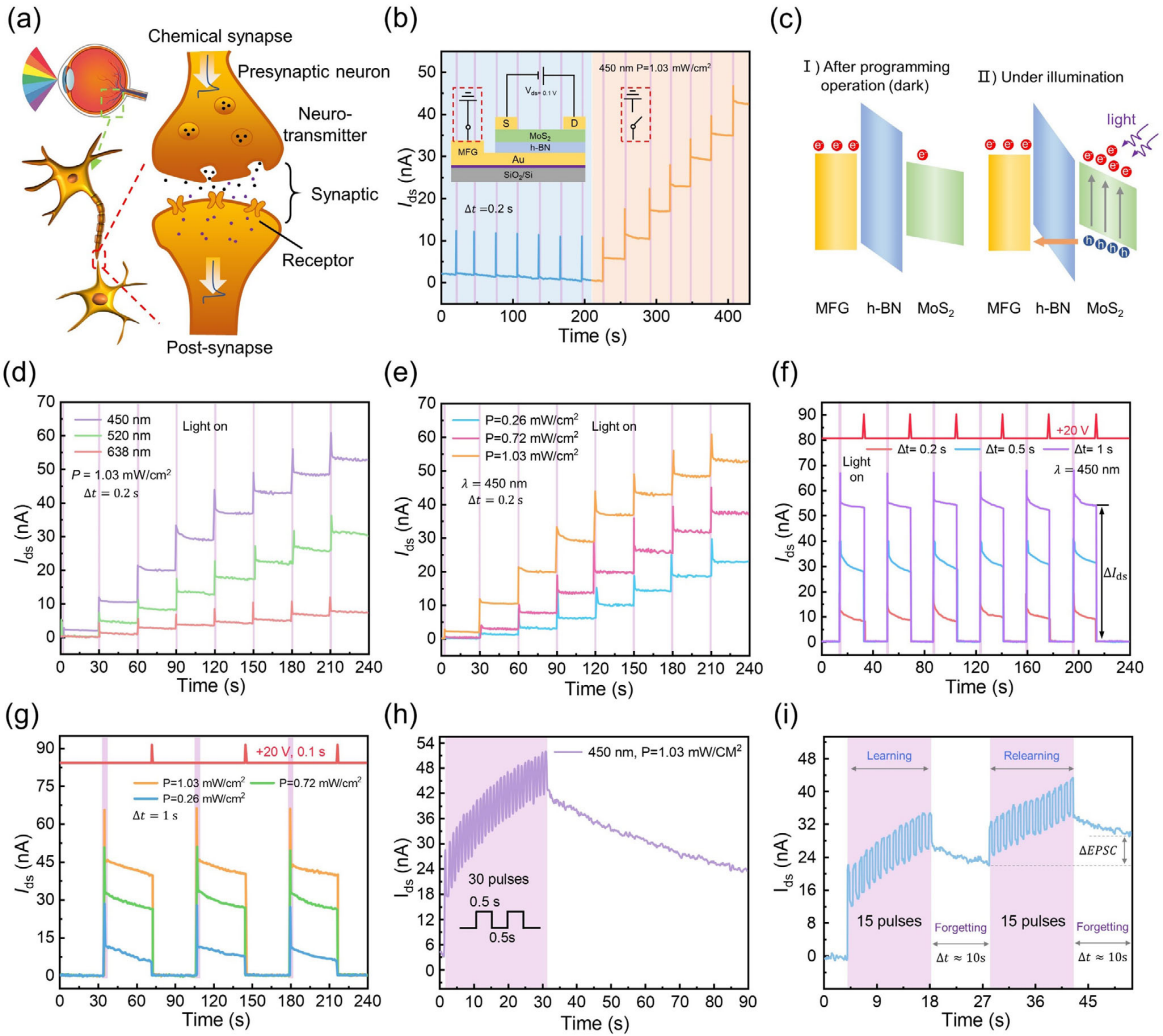
Characterization of optically induced non‐volatile memory and brain‐inspired optoelectronic synaptic functions in the MFG‐FET. (a) Schematic representation of visual signal acquisition and synaptic transmission in a biological visual system. (b) Time‐dependent photoresponse curves of the MFG‐FET with the MFG in grounded and floating states, respectively. (c) Energy band diagrams illustrating the charge dynamics of the optoelectronic synapse. (d) Wavelength‐dependent photocurrent response under pulsed illumination (pulse duration: 0.2 s; period: 30 s; *P* = 1.03 mW/cm^2^; *V*_ds_ = 0.1 V, the same below). (e) Time‐dependent photocurrent with different incident laser powers. (f) Pulse‐duration‐dependent optical modulation of the channel current under 450 nm illumination, followed by a back‐gate pulse (+20 V, 0.1 s) for current initialization. (g) Light‐intensity dependent modulation of the channel current under 450 nm illumination, with back‐gate pulses (+20 V, 0.1 s) applied between optical stimulation cycles. (h) LTP behavior induced by continuous light pulses. (i) The “learning‐forgetting‐relearning” process triggered by multiple light pulses.

In the biological visual system, optical signals are first captured by the retina and then converted into neural signals that are transmitted through interconnected synapses for higher‐order processing (Figure 4a). Inspired by this process, the MFG‐FET was designed to emulate visual synaptic behavior, in which optical pulses serve as presynaptic stimuli and *I*_ds_ represents the postsynaptic current. The visible‐light absorption of multilayer MoS_2_ enables optical sensing, while charge storage in the MFG supports persistent conductance modulation. The wavelength‐dependent photoresponse of the device was first examined over the range of 405–780 nm, and the strongest response was observed at 450 nm (Figure S9).

To verify that the non‐volatile optoelectronic synaptic response originates from charge storage in the MFG rather than from intrinsic defects in MoS_2_ or interfacial traps, an in situ electrical switching experiment was performed. In the grounded‐MFG configuration, optical pulses produced only volatile photocurrent spikes that decayed rapidly after illumination was removed (Figure 4b, blue region; Figure S10). When the MFG was switched to an electrically floating state, the same optical pulses generated a stepwise and retained increase in channel conductance (Figure 4b, orange region). This reversible transition from transient photodetection to non‐volatile optical memory confirms that the MFG acts as the dominant charge‐storage reservoir for the synaptic response.

Before each optical stimulation sequence, the device was initialized to a low‐conductance state by a programming gate pulse, corresponding to an electron‐rich MFG after programming (Figure 4c‐I). Under illumination, electron‐hole pairs are photogenerated in the MoS_2_ channel. Considering the polarity of the optical response, the initial floating‐gate charge state, and the floating‐/grounded‐gate control experiments, we attribute the persistent conductance modulation predominantly to photoinduced modification of the charge stored in the Au floating gate. A possible microscopic pathway is that a fraction of the photogenerated holes undergoes field‐assisted transfer across the h‐BN barrier and partially compensates the programmed electrons in the Au floating gate (Figure 4c‐II). Accordingly, the stepwise light‐induced conductance increase corresponds to partial optical erasure of the preprogrammed and negatively charged Au floating gate [44].

The wavelength‐ and power‐dependent excitatory postsynaptic current (EPSC) responses were then examined to evaluate the optoelectronic synaptic functionality of the MFG‐FET. Under periodic optical pulses with a fixed power density of 1.03 mW/cm^2^, a pulse duration of 0.2 s, and a period of 30 s, the photocurrent increases cumulatively at wavelengths of 450, 520, and 638 nm (Figure 4d). The EPSC amplitude decreases as the wavelength increases, indicating more efficient optical stimulation under higher photon‐energy illumination. During the dark intervals, the current remained above its initial baseline rather than fully relaxing, evidencing persistent charge modulation in the MFG. At a fixed wavelength of 450 nm, increasing the power density from 0.26 to 1.03 mW/cm^2^ enhanced both the EPSC amplitude and the conductance accumulation rate (Figure 4e). These results demonstrate that the synaptic weight can be continuously regulated by both the photon energy and the optical power density.

A combined light‐induced partial erasure and electrical reprogramming scheme was further implemented at *V*_ds_ = 0.1 V under 450 nm illumination to demonstrate controllable multilevel conductance states. As shown in Figure 4f, increasing the optical pulse duration from 0.2 to 1 s produced progressively larger increments in channel current, reflecting time‐dependent photogenerated charge accumulation and redistribution in the MFG structure. After each light‐induced partial erasure event, a positive back‐gate pulse (+20 V, 1 s) was applied to reprogram the Au floating gate into the same electron‐rich, low‐conductance initial state before the next optical stimulation, enabling repeatable optical modulation cycles. When the optical pulse duration was fixed and the incident power density was varied from 0.26 to 1.03 mW/cm^2^, the device produced clearly distinguishable conductance levels during optical stimulation (Figure 4g). This optoelectronic modulation demonstrates controllable synaptic‐weight tuning by optical pulse duration and power density.

To simulate the LTP behavior of biological synapses, we applied a series of continuous light pulses (λ = 450 nm) to the MFG‐FET. As illustrated in Figure 4h, under a stimulation sequence of 30 laser pulses (1 Hz repetition rate, 50% duty cycle), *I*_ds_ exhibited a staircase‐like enhancement. The nonlinear conductance evolution arises from cumulative optical modulation of the charge state in the MFG, each light pulse generates carriers in MoS_2_ and incrementally changes the floating‐gate charge state through the h‐BN tunneling barrier, thereby increasing the channel conductance. After the stimulation sequence, the potentiated conductance state showed only slow relaxation, consistent with non‐volatile electrostatic confinement in the deep‐potential MFG. Furthermore, the optical potentiation displays pronounced spectral selectivity (Figure S11). While 520 nm illumination successfully emulates progressive LTP, longer wavelengths (638 and 780 nm) fail to elicit discernible potentiation. The wavelength dependence likely results from the combined effects of the optical absorption of multilayer MoS_2_ and photoassisted charge transfer across the h‐BN barrier. Shorter‐wavelength illumination generally produces a larger retained conductance modulation, with the maximum response observed at 450 nm [45].

To quantitatively characterize the temporal dynamics of the optical response, a time‐resolved measurement was performed under continuous 450 nm illumination. Biexponential fitting yielded fast and slow rise time constants of 0.49 and 9.62 s, respectively, and fast and slow decay time constants of 0.96 and 141.65 s, respectively (Figure S12). The faster components are associated with the prompt device‐level photoresponse and possible shallow‐trap dynamics, whereas the slower components are consistent with interfacial charge trapping/detrapping and floating‐gate‐mediated charge relaxation [46, 47].

Beyond basic synaptic plasticity, the device emulated a biological memory‐consolidation process that includes learning, forgetting, and relearning (Figure 4i). During the initial learning phase, 15 consecutive optical pulses increased *I*_ds_ to approximately 34.1 nA. During the following 10 s dark interval, which represents spontaneous forgetting, the current decayed to about 21.8 nA. In the re‐learning phase, the current recovered to the previously learned level after only approximately four optical pulses and further increased to about 34.8 nA by the end of the 15‐pulse sequence. After a second 10 s dark interval, the current remained at approximately 29.2 nA, showing a smaller decay than after the first learning process. The faster reacquisition and improved retention after repeated stimulation indicate history‐dependent memory consolidation, analogous to review‐enhanced learning in biological synapses.

**FIGURE 5:**
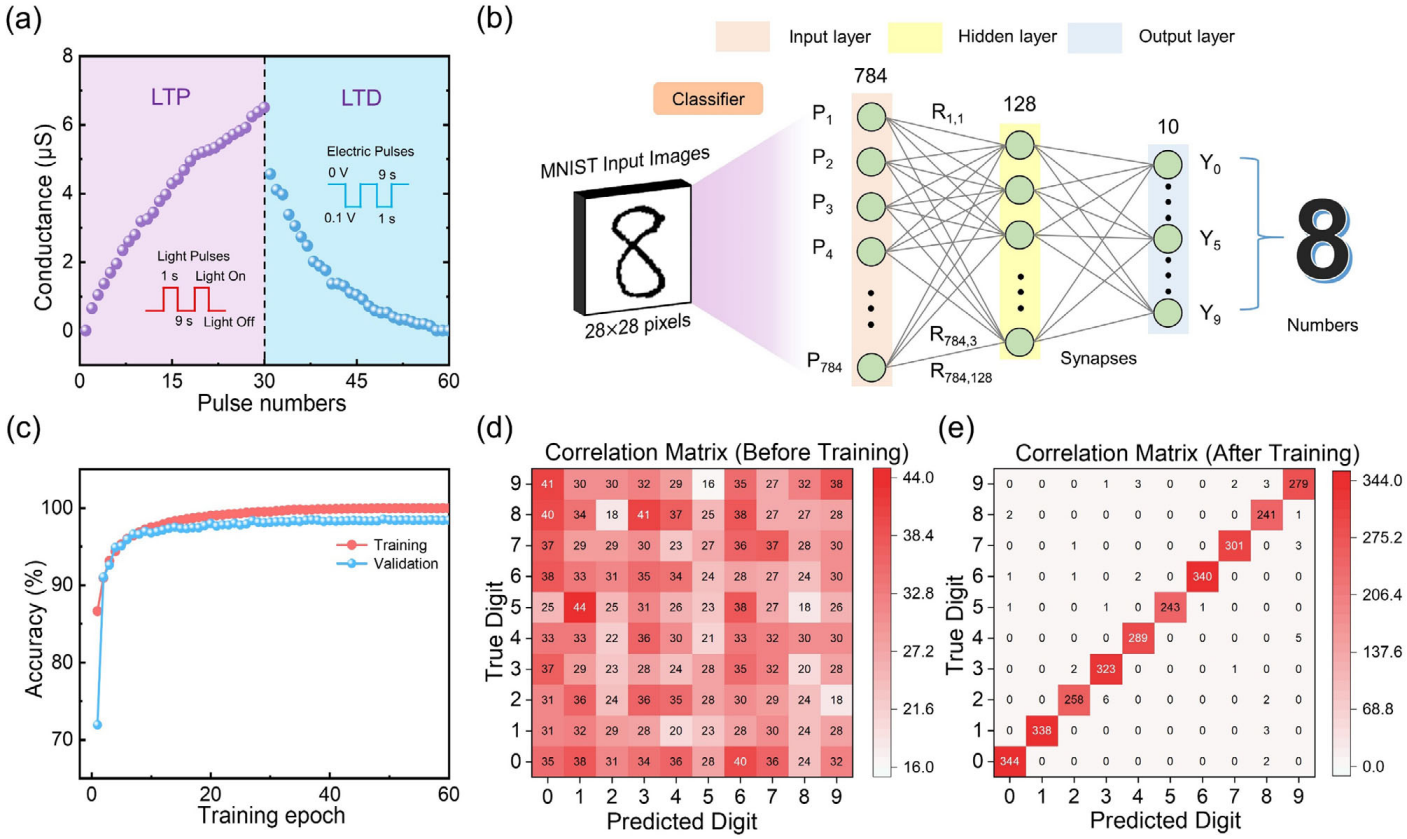
Neuromorphic computing based on the MFG‐FET. (a) Light‐induced LTP and voltage‐induced LTD behavior. (b) Schematic architecture of the simulated three‐layer HOENN based on the device. (c) Evolution of accuracy over time during neural network training; both the training and validation sets exhibit rapid convergence. (d) Confusion matrix before training, demonstrating a random prediction distribution across different digit categories in the model's untrained state. (e) Confusion matrix after training, where predictions are primarily concentrated along the diagonal, indicating that the model achieves high recognition accuracy.

To evaluate the feasibility of the proposed optoelectronic synaptic transistor for neuromorphic computing, we constructed a hybrid optoelectronic neural network (HOENN) modeled on its hardware conductance modulation to perform a handwritten digit recognition task. As illustrated in Figure 5a, the device exhibits stable LTP under optical stimulation and LTD under electrical pulses. This continuous and reversible dual‐mode modulation confirms its robust capability for precise synaptic weight updates. To establish the connection between the experimental data of the device and the neural‐network simulation, the channel current measured at *V*_ds_ = 0.1 V after each stimulation pulse was converted into conductance according to *G*  = *I*_ds_ /*V*_ds_. The experimentally measured LTP and LTD conductance trajectories were fitted and normalized as ω (*n*) = [*G*(*n*) − *G*_min_] /[*G*_max_ − *G*_min_], where *G*_min_ and *G*_max_ are the minimum and maximum conductance values extracted from the experimental curves. During network training, each positive or negative gradient update corresponds to one equivalent pulse along the fitted LTP or LTD trajectory, respectively. In this manner, the experimentally observed nonlinear conductance modulation is incorporated into the synaptic‐weight update process.

Based on this plasticity, the HOENN was configured as a three‐layer network comprising 784 input neurons, 128 hidden neurons, and 10 output neurons tailored for decimal digit classification. We trained the network using the MNIST dataset superimposed with 10% white noise. As shown in Figure 5c, the recognition accuracy on both training and validation sets increases rapidly and converges within a short duration, reflecting the high learning efficiency facilitated by the device's reliable weight modulation. The network ultimately achieves an outstanding accuracy of 99.9% on the training set and 98.4% on the test set. This classification performance is visually corroborated by the confusion matrices. While the untrained network yields a stochastic, disordered distribution (Figure 5d), the post‐training matrix (Figure 5e) demonstrates that predicted values are highly concentrated along the principal diagonal. This distinct transition from initial random uncertainty to organized, category‐specific precision confirms the model's ability to distinguish digit categories with high fidelity, performing on par with state‐of‐the‐art neuromorphic systems [48, 49].

To further visualize the inference capability of the MFG‐FET synaptic network, representative recognition results from the MNIST test set are presented in Figure S13. The network, utilizing the experimentally extracted weight‐update characteristics, successfully identifies diverse handwritten digits with a consistent classification confidence of 100%. These results indicate that the opto‐synaptic transistor not only emulates biological synaptic plasticity but also effectively supports neural network training and inference, providing a robust hardware foundation for efficient neuromorphic visual computing.

As summarized in Table 1, recently reported graphene floating‐gate devices exhibit important advantages in low‐voltage and fast operation, multilevel storage, dual‐mode conduction, and reconfigurable functions. Therefore, Au should not be regarded as universally superior to graphene. The Au floating gate offers its relatively high work function, high density of states, and chemical stability, which are expected to support stable charge confinement. The h‐BN/Au electron‐confinement barrier is estimated to be 2.8–3.1 eV, which is higher than that of intrinsic graphene/h‐BN of 2.3–2.6 eV. Correspondingly, the MoS_2_/h‐BN/ Au device presents the high‐performance metrics of an ON/OFF ratio of approximately 10^5^, retention exceeding 3000 s, endurance over 1600 cycles, optoelectronic modulation, and reproducible operation after 200 days of ambient atmospheric storage. These results indicate that the Au and graphene floating gates provide complementary advantages.

**TABLE 1:** Summary of the reported floating gate devices.

Device structure	ON/OFF	Memory window *V*_G, max_ ratio	Operation voltage V_set_/V_reset_	Retention time	Endurance	Optical modulation	Reference
ReS_2_/h‐BN/Gr	10^7^	94.5%	+40/−40 V	>1100 s	>1000 cycles	Yes	[50]
ReS_2_/h‐BN/Gr (Top floating gate)	∼10^6^	63%	+30/−30 V	>10 000 s	>1000 cycles	Yes	[19]
BP/Al_2_O_3_/BP	∼10^3^	—	+5/−5 V	>100 s	500 cycles	No	[51]
MoS_2_/h‐BN/Te	∼10^8^	95.1%	+12/−12 V	>4000 s	>4000 cycles	Yes	[44]
MoS_2_/h‐BN/PdSe_2_	10^7^	75.5%	+40/−40 V	>10 000 s	>1000 cycles	Yes	[52]
MoS_2_/h‐BN/Gr	∼10^5^	89.83%	+9/−7 V	>1500 s	>1000 cycles	No	[53]
MoS_2_/h‐BN/Gr	10^5^	60%	+50/−50 V	>6000 s	>100 cycles	No	[25]
SnS_2_/h‐BN/ Au	10^8^	87.5%	+40/−40 V	>10^5^ s	>15000 cycles	No	[34]
ReSe_2_/h‐BN/Gr	>10^4^	>70%	+60/‐60 V	>1000 s	>3000 cycles	Yes	[54]
MoTe_2_/h‐BN/Gr	>10^4^	>65%	+60/−60 V	>1000 s	>1000 cycles	No	[55]
ReS_2_/h‐BN/Gr	10^5^	81.82%	+8/−8 V	>1000 s	>1000 cycles	Yes	[56]
MoS_2_/h‐BN/Au	10^5^	81.3%	+20/−20 V	>3000 s	>1600 cycles	Yes	This work

## 3 Conclusion

In summary, we have demonstrated a stable non‐volatile optoelectronic synaptic FET based on a MoS_2_/h‐BN/Au deep‐potential MFG architecture. By integrating a high work function Au nanofilm as a discrete charge‐storage layer, we mitigate environmental sensitivity and Fermi‐level fluctuation issues commonly encountered in Gr‐based floating gates. The deep potential well enables modulated charge‐tunneling dynamics, yielding a memory window of 97.6 V. The device exhibited two‐state data retention exceeding 3000 s and cycling endurance over 1600 programming/erasing cycles. Separately, reproducible programming/erasing functionality was retained after 200 days of ambient storage. Furthermore, electrical switching experiments help decouple the dominant role of floating‐gate storage from intrinsic channel or interfacial defects. Using these reliable weight updates, hardware‐algorithm co‐simulations achieved a recognition accuracy of 98.4% in the MNIST task. This work underscores the potential of the MFG architecture for multimodal information storage and provides a hardware paradigm for integrated neuromorphic vision platforms.

## 4 Methods

### 4.1 Device Fabrication

The complete fabrication flow of the MoS_2_/h‐BN/Au optoelectronic synaptic FET is schematically illustrated in Figure S1. Initially, SiO_2_/p+Si substrates were sequentially cleaned by ultrasonication in acetone, ethanol, and deionized water for 10 min each to eliminate surface contaminants. Following the spin‐coating of photoresist (AZ 5214), the MFG electrodes were patterned via ultraviolet photolithography (SUSS MA6, 365 nm). A Cr/Au film (10/30 nm) was subsequently deposited utilizing thermal evaporation, followed by a standard acetone lift‐off process to remove the residual resist. For the vdW heterostructure assembly, multilayer h‐BN and MoS_2_ flakes (Shanghai Onway Technology Co., Ltd) were mechanically exfoliated onto polydimethylsiloxane (PDMS) stamps. The selected two‐dimensional flakes were deterministically transferred onto the pre‐fabricated MFG using a micromanipulator transfer stage, and the PDMS carrier was released by heating to 80°C. To define the source/drain contacts, poly(methyl methacrylate) (PMMA) resist was spin‐coated onto the sample surface. The contact regions were patterned using electron beam lithography (EBL) integrated with a scanning electron microscope (SEM, ZEISS Sigma 300). After development, Cr/Au (10/40 nm) contact electrodes were deposited strictly via thermal evaporation, and the final devices were obtained after the subsequent lift‐off procedure.

### 4.2 Measurements

Morphological and structural characterizations of the fabricated heterostructure devices were initially conducted using optical microscopy (Olympus, BX41M) to evaluate the spatial alignment and surface integrity of the MFG and the transferred two‐dimensional flakes. Atomic force microscopy (AFM, Bruker MM8) was subsequently employed to precisely determine the physical thickness and topographical uniformity of the exfoliated h‐BN and MoS_2_ layers. Raman spectroscopy was performed using a Shamrock 500i spectrometer system. The electrical transport properties and optoelectronic synaptic functions were systematically evaluated under ambient conditions using a semiconductor parameter analyzer (Keithley 4200A‐SCS). All the above tests were conducted at room temperature and under ambient conditions.

## Author Contributions

**Shuo Lei**: methodology, formal analysis, investigation, validation, Writing – original draft, Writing – review and editing, project administration, conceptualization, data curation. **Shichen Zhang**: formal analysis, software. **Zhipeng Zheng**: software, formal analysis. **Miao Cai**: writing – review and editing, formal analysis, methodology. **Shun Tang**: validation. **Xuguang Guo**: writing – review and editing, data curation, supervision. **Yiming Zhu**: funding acquisition, supervision.

## Conflicts of Interest

The authors declare no conflicts of interest.

## Supporting information




**Supporting File**: advs78038‐sup‐0001‐SuppMat.docx.

## Data Availability

All data needed to evaluate the conclusions in the paper are present in the paper and the Supporting Information.
