# Supplementary material for: Highly Stable and Non‐Volatile Optoelectronic Synapses for Sensing, Memory, and Computing Enabled by a Deep‐Potential Floating Gate
Source: Adv Sci (Weinh). 2026 Sep 27:e78038. Online ahead of print. doi: 10.1002/advs.78038 (PMC13616391; doi:10.1002/advs.78038)
Supplement: Supplementary file 1 [file ADVS-9999-e78038-s001.docx]

Supporting Information for

**Highly Stable and Non-volatile Optoelectronic Synapses for Sensing, Memory, and Computing Enabled by a Deep-Potential Floating Gate**

*Shuo Lei et al.*

Corresponding authors: Xuguang Guo, [xgguo@usst.edu.cn](mailto:xgguo@usst.edu.cn) and Yiming Zhu, [ymzhu@usst.edu.cn](mailto:ymzhu@usst.edu.cn)

**Figure S1**. Fabrication process of the MFG optoelectronic synaptic transistor.

The MFG-FET was fabricated on a SiO_2_/Si substrate. The substrate was first cleaned using acetone, alcohol and deionized water cleaning procedures to remove organic and particulate contaminants. A photoresist layer was then spin-coated onto the substrate, followed by ultraviolet lithography through a predefined mask to pattern the electrode regions. After development, Cr/Au electrodes were deposited by thermal evaporation, and a lift-off process was performed to obtain well-defined metal contacts. Subsequently, few-layer MoS_2_ and h-BN flakes were mechanically exfoliated and transferred onto the pre-patterned substrate using a polydimethylsiloxane (PDMS)-assisted dry transfer technique, ensuring clean interfaces and precise alignment. The h-BN layer serves as a dielectric spacer, while MoS_2_ acts as the photoactive channel material. To further define the geometry of the device, a layer of polymethylmethacrylate (PMMA) resist is spin-coated, and then the channel and contact areas are patterned through electron beam lithography (EBL). After development, the final device structure was obtained through thermal evaporation and a lift-off process, completing the fabrication of the optoelectronic synaptic transistor.

**Figure S2.** Output curves of the devices with a) no-floating gate and b) metal semi-floating gate structure measured under different gate voltages.

**Figure S3.** Thickness-dependent memory window modulation enabled by h-BN dielectric layer. a), b) Optical images of devices incorporating h-BN flakes with different thicknesses. c), d) Atomic force microscopy height profiles along the dashed lines in a) and b), indicating h-BN thicknesses of ~19 nm and ~40 nm, respectively. e), f) Corresponding transfer characteristics under forward and reverse gate voltage sweeps for devices.

To investigate the role of the dielectric layer in the memory behavior of the device, we fabricated MFG devices with different h-BN thicknesses and performed a comparative analysis. Optical images of two representative devices are shown in Figure S3a and b, and the corresponding AFM height profiles confirm h-BN thicknesses of approximately 19 nm and 40 nm, respectively. The transfer characteristics under bidirectional gate voltage sweeps are presented in Figure S3e and f. Both devices exhibit hysteresis behavior, indicating the presence of charge trapping and de-trapping processes. Notably, the memory window decreases with increasing h-BN thickness: the thinner h-BN (~19 nm) results in a larger threshold voltage shift, whereas the thicker h-BN (~40 nm) exhibits a smaller hysteresis window. This behavior can be attributed to the modulation of charge injection by the dielectric thickness^[1]^. A thicker h-BN layer increases the tunneling barrier thickness for carrier injection from the channel into the floating gate, thereby significantly suppressing the charge injection efficiency. Consequently, the stored charge density is reduced, leading to a weaker modulation of the channel conductance. These results demonstrate that the thickness of the h-BN dielectric layer plays a critical role in device design, requiring a trade-off between charge injection efficiency and memory retention capability.

**Figure S4.** Transient characteristics of MFG-FET during writing and erasing. a) Transient characteristic during writing and erasing with pulse amplitude of 50 V. b) Transient characteristic during writing and erasing with pulse peak of 20 V. c) Transient characteristic during writing and erasing with pulse peak of 10 V. d) Electrical erasing operation, electrical programming operation, and reading of the erasing/programming state of the non-volatile memory at *V*_ds_= 0.1 V.

**Figure S5.** Post-storage programming/erasing characteristics of the MFG-FET measured after 200 days of storage under ambient conditions using nominal 0.02 s gate-voltage pulses.

Channel-current stability of the MFG-FET measured at *V*_ds_=0.1 V after the device had been stored under laboratory ambient conditions for 200 days. Following storage, erasing and programming gate-voltage sweeps to -20 and +20 V, respectively, were applied with a nominal duration of 0.02 s. The resulting high- and low-conductance states remained clearly separated during the subsequent measurement interval, with an ON/OFF ratio of approximately 10^5^. These results demonstrate reproducible programming/erasing operation using nominal 0.02 s gate-voltage pulses after 200 days of ambient storage.

**Figure S6.** a) Electrical band alignment used in the numerical calculation. b) Experimental transfer characteristic (symbols) and calculated curves (solid lines) for the complete -60 V to +60 V and back -60 V gate-voltage loop.

A history-dependent electrostatic model ^[2,3]^ was adopted to explore the carrier transfer between the floating gate and the channel. Because the floating-gate Au layer has a high density of states, its quantum capacitance was assumed to be much larger than the geometrical capacitances, and the Fermi-level shift of the metal floating gate was neglected. As shown in Figure S6a), there are expressions,

$$\begin{aligned} \left\{ \begin{aligned} \Delta n_{C}=\frac{{-C}_{\mathrm{BN}}\varphi_{\mathrm{FG}}}{e} \\ V_{G}=\varphi_{G} \\ \Delta n_{m}=-\Delta n_{C}+\frac{C_{\mathrm{OX}}\left( \varphi_{\mathrm{FG}}-\varphi_{G} \right)}{e} \end{aligned} \right..\#\left( S1 \right) \end{aligned}$$

All the parameters are listed and explained in Table S1.

**Table S1.** Parameters in Figure S6 and Equations (S1).

| Parameter | Description | Value |
| --- | --- | --- |
| $\chi_{C}$ $W_{M}$, $\chi_{\mathrm{BN}}$, $\chi_{Si}$ and $\chi_{\mathrm{OX}}$ | Affinity energies of MoS_2_, Au, h-BN, Si and SiO_2_ | Parameters |
| $\varphi_{G}$ and $\varphi_{\mathrm{FG}}$ | Channel built-in potential and floating gate | Unknown variables |
| $\Delta n_{C}$ and $\Delta n_{m}$ | Back-gate-induced carrier concentration variations of MoS_2_, and MFG respectively | Unknown variables |
| $C_{\mathrm{OX}}$ and $C_{\mathrm{BN}}$ | Capacities of SiO_2_ and h-BN layer, $\frac{\varepsilon_{\mathrm{OX}}}{d_{\mathrm{OX}}}$and $\frac{\varepsilon_{\mathrm{BN}}}{d_{\mathrm{BN}}}$ with ε the dielectric constant and d the layer thickness | Predefined parameters |

In this model, the carrier transfer between the floating gate and the channel is dependent on the voltage applied on the h-BN layer. When the voltage applied across the h-BN layer is between *V*_NT_ (<0 V) and *V*_PT_ (>0 V), both the FN tunneling and the direct tunneling are prohibited, and the device is in the capacitive coupling state that there is no carrier transfer between the floating gate and the channel. Once the voltage applied across the h-BN layer reaches up to *V*_NT_ or *V*_PT_, the FN tunneling channel is opened, and due to the screening effect of excess carriers stored in the floating gate layer or in the channel, the voltage across the h-BN layer is pinned at *V*_NT_ or *V*_PT_, which is defined as the feedback FN tunneling. By using this model, the experimental bidirectional transfer curves are numerically fitted with *V*_NT_ and *V*_PT_ as two free parameters. The measured geometry and dielectric constants (*d*_h-BN_=11.4 nm, *d*_ox_=300 nm, $\varepsilon_{h-BN}$=3.5, $\varepsilon_{\mathrm{OX}}$=3.9, *V*_ds_=0.1 V and channel length to width ratio=2.0) were fixed. Using the parameters in Table S1 and the fitting parameters *V*_NT_=-0.37 V and *V*_PT_=0.75 V, the calculated -60 V to +60 V and back -60 V bidirectional loop agrees well with the measured transfer curve (Figure S6b), with a mean logarithmic root mean squared error of 0.194 dec, which indicates that the capacitive and the feedback FN tunneling coupling mechanisms capture the main physical process of the device and FN tunneling plays a dominate role on the memory behavior of our device.

**Note S1.** Fowler-Nordheim tunneling analysis.

To further evaluate the field-assisted charge-transfer process through the h-BN barrier, the measured current-voltage characteristic between the Au floating gate and the channel of an Au/h-BN/MoS_2_ was analyzed using the FN formalism^[4]^. Following the conventional treatment of a trapezoidal barrier under a sufficiently high electric field, the tunneling current can be expressed as,

$$\begin{aligned} I=AV^{2}exp\left( \frac{-B}{V} \right),\#\left( S2 \right) \end{aligned}$$

where *I* is the measured tunneling current, *V* is the applied voltage, and *A* and *B* are the voltage-independent prefactor and exponential coefficient, respectively. In the ideal one-dimensional FN tunneling approximation, these coefficients are given by,

$$\begin{aligned} A=\frac{A_{eff}q^{3}m}{8\pi h\varphi_{B}d^{2}m^{*}},B=\frac{8\pi\sqrt{2m^{*}}\varphi_{B}^{\frac{3}{2}}d}{3hq},\#\left( S3 \right) \end{aligned}$$

where *A*_eff_ is the effective tunneling area, *q* is the elementary charge, *h* is Planck's constant, *m* is the free-electron mass, *m** is the effective tunneling mass, *d* is the h-BN thickness, and $\varphi_{B}$ is the effective barrier height. Equation (S2) can therefore be linearized as,

$$\begin{aligned} \ln\left( \frac{|I|}{V^{2}} \right)=\ln A-B\left( \frac{1}{V} \right).\#\left( S4 \right) \end{aligned}$$

Accordingly, a plot of ln(*I/V*^2^) versus 1/*V* should contain a negative linear region when the FN tunneling is the dominant mechanism. The voltage range from 6.52 to 10.28 V was selected because it covers the post-knee monotonic current increase and terminates immediately before the abrupt current step at 10.32 V. The low-voltage noise-floor region and the post-step regime were excluded.

**Figure S7.** FN tunneling analysis of carrier transport across the h-BN barrier. a) Measured *I-V* characteristic plotted on a semilogarithmic current scale; the shaded area denotes the 6.52-10.28 V voltage range used for the FN tunneling analysis. b) Plot of ln(*|I|/V^2^*) versus 1/*V* for the selected high-field region. The red line is an unweighted least-squares fit to the experimental data.

As shown in Figure S7b), the transformed data exhibit an approximately linear negative trend over the selected high-field region. The least-square fit gives,

$$\begin{aligned} \ln\left( \frac{|I|}{V^{2}} \right)=-9.256945-115.156456\left( \frac{1}{V} \right),R^{2}=0.98065,\#\left( S5 \right) \end{aligned}$$

for 95 data points. The fitted slope and intercept are -115.156 ± 1.677 V and -9.257 ± 0.205, respectively. Therefore, *B*=115.156 V and *A*=exp(intercept)=9.54465×10^-5^ A/V^2^. The approximately linear relationship between ln(*I/V*^2^) and 1/*V* (*R*^2^=0.9807) is consistent with an FN-type tunneling contribution in the high-field regime.

**Figure S8.** Energy band diagrams of the device under different programming and erasing pulse amplitudes.

Figure S8 illustrates the energy-band evolution of the MoS_2_/h-BN/Au floating-gate architecture under varying applied gate-voltage pulses. The mechanism for the discrete multilevel memory states is governed by electric-field-modulated charge tunneling^[5]^. By modulating the amplitude of the back-gate voltage pulses, the electric field between the back gate and the channel is tuned, which changes the effective tunneling barrier across the h-BN dielectric layer. Under different barrier conditions, the amount of charge transferred between the MoS_2_ channel and the MFG varies, resulting in distinct channel conductance states and enabling multilevel memory behavior. During programming with a positive back-gate pulse, electrons tunnel from the MoS_2_ channel into the MFG, depleting the channel and decreasing the readout current. During erasing with a negative back-gate pulse, the reversed field drives electron back-tunneling from the MFG to the MoS_2_ channel, restoring the channel carrier density and increasing the readout current.

**Figure S9.** Channel photocurrent response to different light wavelengths ranging from 405 to 780 nm.

The photovoltaic response was experimentally characterized under zero gate bias and global uniform irradiation at *V*_ds_ = 0.1 V. The device was excited using collimated laser beams across a wavelength spectrum from 405 nm to 780 nm, with the optical power density precisely calibrated at 1.03 mW/cm^2^. The resulting photocurrent-time (*I*_ds_-*T*) curves reveal a peak responsivity at a wavelength of 450 nm. For the same power density, the 450-nm illumination provides approximately 11% more photons than the 405-nm illumination, and at the same time, the 450-nm laser photon energy of 2.76 eV lies near the C-exciton/band-nesting high absorption region of MoS_2_, which are responsible for the higher optical programming efficiency. This observation is highly concordant with the intrinsic absorption spectrum of multilayer MoS_2_ ^[6]^.

**Figure S10.** Photo-induced current response of the NFG region under periodic light pulse illumination. In the absence of a charge-trapping layer, the device exhibits purely volatile transient photocurrents with no retention or memory characteristics.

**Figure S11**. Wavelength-dependent dynamic photoresponse and relaxation behavior. Time-resolved channel current under pulsed illumination at wavelengths of a) 520 nm, b) 638 nm, and c) 780 nm, respectively, with a fixed power density of 1.03 mW/cm^2^. The shaded regions indicate the illumination period.

To investigate the spectral response of synaptic weight modulation, a sequence of continuous optical pulses was applied to the MoS_2_/h-BN/Au MFG-FET at a constant source-drain voltage of 0.1 V, while the channel current variations were monitored in real time. Under a stimulus train consisting of 30 laser pulses with a repetition frequency of 1 Hz and a 50% duty cycle, the device was irradiated at wavelengths including 450, 520, 638, and 780 nm. As the incident wavelength increases, the photocurrent increment gradually decreases, indicating pronounced spectral selectivity in the optical synaptic response. Within the proposed mechanism, short-wavelength illumination may facilitate photoassisted charge transfer across the h-BN barrier and modify the net charge stored in the floating gate, thereby producing a persistent electrostatic modulation of the MoS_2_ channel conductance. In contrast, longer-wavelength illumination produces a weaker retained conductance modulation because the lower photon energy and reduced effective absorption in MoS_2_ decrease the probability of field-assisted carrier transfer across the h-BN barrier and, consequently, the modulation of the MFG charge state.

**Figure S12.** Time-resolved optical response and characteristic-time analysis of the MFG-FET.

The temporal response under continuous 450 nm illumination was quantitatively analyzed using biexponential functions because both the rise and decay curves exhibit distinguishable fast and slow components. The rise transient was fitted using,

$$\begin{aligned} I_{\mathrm{rise}}\left( t \right)=I_{\mathrm{dark}}+A_{r1}\left[ 1-e^{-\frac{t-t_{\mathrm{on}}}{\tau_{r1}}} \right]+A_{r2}\left[ 1-e^{-\frac{t-t_{\mathrm{on}}}{\tau_{r2}}} \right],\#\left( S6 \right) \end{aligned}$$

where $I_{\mathrm{dark}}=0.272 nA$, $A_{r1}=26.25 nA$, and $A_{r2}=25.44 nA$. The extracted fast and slow rise time constants were $\tau_{r1}=0.49 s$ and $\tau_{r2}=9.62 s$, respectively, with $R^{2}=0.9963$.

The post-illumination decay was fitted using,

$$\begin{aligned} I_{\mathrm{decay}}\left( t \right)=I_{\mathrm{dark}}+A_{d1}e^{-\frac{t-t_{\mathrm{off}}}{\tau_{d1}}}+A_{d2}e^{-\frac{t-t_{\mathrm{off}}}{\tau_{d2}}},\#\left( S7 \right) \end{aligned}$$

where $A_{d1}=14.81 nA$ and $A_{d2}=33.88 nA$. The extracted fast and slow decay time constants were $\tau_{d1}=0.96 s$ and $\tau_{d2}=141.65 s$, respectively, with $R^{2}=0.9708$.

For the independent threshold-based analysis, the response was normalized according to,

$$\begin{aligned} R\left( t \right)=\frac{I\left( t \right)-I_{\mathrm{dark}}}{I_{\mathrm{peak}}-I_{\mathrm{dark}}}.\#\left( S8 \right) \end{aligned}$$

The 10%-90% rise time and 90%-10% decay time were determined to be approximately 14.70 s and 188.09 s, respectively.

**Figure S13.** Representative inference results of the MNIST handwritten digit recognition task.

A selection of test images processed by the simulated artificial neural network (ANN) based on the experimental synaptic weight updates. Each sub-image displays the original input digit, where “P” denotes the predicted label assigned by the network and “C” represents the corresponding classification confidence. The successful identification of all digits with a confidence score of 1.00 corroborates the exceptional reliability and high accuracy of the hardware-algorithm co-design.

**Reference**

1. W. Li, J. Li, Y. Chen, et al., “Demonstration of Nonvolatile Storage and Synaptic Functions in All-Two-Dimensional Floating-Gate Transistors Based on MoS2 Channels,” *ACS Applied Electronic Materials* *5*, no. 8 (2023): 4354–4362, https://doi.org/10.1021/acsaelm.3c00595.

2. J. Fu, H. Shi, M. Cai, et al., “Non‐Volatile and Gate‐Controlled Multistate Photovoltaic Response in WSe_2_ /h‐BN/Graphene Semi‐Floating Gate Field‐Effect Transistors,” *Advanced Optical Materials* *12*, no. 21 (2024): 2400638, https://doi.org/10.1002/adom.202400638.

3. T. Sasaki, K. Ueno, T. Taniguchi, K. Watanabe, T. Nishimura, and K. Nagashio, “Material and Device Structure Designs for 2D Memory Devices Based on the Floating Gate Voltage Trajectory,” *ACS Nano* *15*, no. 4 (2021): 6658–6668, https://doi.org/10.1021/acsnano.0c10005.

4. H. Lu, Y. Wang, X. Han, and J. Liu, “An Ultrafast Multibit Memory Based on the ReS2/h-BN/Graphene Heterostructure,” *ACS Nano* *18*, no. 34 (2024): 23403–23411, https://doi.org/10.1021/acsnano.4c06642.

5. S. H. Kim, S.-G. Yi, M. U. Park, C. Lee, M. Kim, and K.-H. Yoo, “Multilevel MoS2 Optical Memory with Photoresponsive Top Floating Gates,” *ACS Applied Materials & Interfaces* *11*, no. 28 (2019): 25306–25312, https://doi.org/10.1021/acsami.9b05491.

6. S. Zhang, M. Cai, Y. Chen, et al., “Switchable Photovoltaic and Synaptic Dual-Mode Behaviors in Asymmetric MoS2 Schottky Phototransistors,” *Photonics Research* (2026), https://doi.org/10.1364/PRJ.589551.
